# Self-rated health is associated with the length of stay at the intensive care unit and hospital following cardiac surgery

**DOI:** 10.1186/1471-2261-14-171

**Published:** 2014-11-29

**Authors:** Zsuzsanna Cserép, Eszter Losoncz, Roland Tóth, Attila Tóth, Boglárka Juhász, Piroska Balog, Péter Vargha, János Gál, Richard J Contrada, Paul RJ Falger, Andrea Székely

**Affiliations:** The School of Ph.D. Studies, Semmelweis University, Budapest, Hungary; The Department of Anesthesia and Intensive Care, Semmelweis University, Budapest, Hungary; The Department of Anesthesia and Intensive Care, Gottsegen György Hungarian Institute of Cardiology, Budapest, Hungary; The Department of Cardiac Surgery of Gottsegen György Hungarian Institute of Cardiology, Budapest, Hungary; The Institute of Behavioral Sciences, Semmelweis University, Budapest, Hungary; The Hungary Heart Center, Semmelweis University, Budapest, Hungary; The Department of Psychology, Rutgers the State University of New Jersey, New Brunswick, New Jersey USA; Department of Psychiatry and Neuropsychiatry, Faculty of Health Medicine and Life Sciences, Maastricht University, Maastricht, The Netherlands

**Keywords:** ICU stay, Happiness, Self-rated health, Cardiac surgery, Risk stratification

## Abstract

**Background:**

Recently, a considerable amount of evidence suggested that anxiety, depression and other psychosocial variables might influence the outcomes of cardiac surgery. This study investigated the relationship between length of stay at the intensive care unit (ICU) and hospital after surgery and different psychosocial variables (e.g. depression, anxiety, self rated health, happiness, satisfaction).

**Methods:**

We enrolled prospective patients who were waiting for elective cardiac surgery (N = 267) and consented to take part in the study. We collected data of sociodemographic, medical and perioperative factors as well as psychosocial questionnaires completed 1.56 days (standard deviation [SD] = 0.7) before surgery. The primary clinical endpoint was an ICU stay of at least 3 days and the secondary was hospital stay of at least 10 days.

**Results:**

Two hundred sixty-seven patients participated in this study. Four patients (1.5%) died in the hospital and 38 patients (14.5%) spent more than 3 days in the ICU and 62 patients (23.2%) spent more than 10 days in the hospital. After controlling for medical and sociodemographic factors, lower self rated health (Adjusted Odds Ratio [AOR]: 0.51, 95% confidence interval [CI]: 0.28-0.95; p = 0.03), lower rate of happiness (AOR: 0.76, 95% CI: 0.59-0.97, p = 0.03), postoperative cardiac failure (AOR: 7.09, 95% CI:1.21-41.54; p = 0.03) and postoperative complications (AOR: 9.52, 95% CI: 3.76-24.11; p < 0.001) were associated with longer ICU stay. More than 10 days of hospital stay was associated with higher occurrence of COPD (AOR 4.56, CI: 1.95-10.67, p < 0.001), NYHA stage (AOR 6.76, CI: 2.57-17.79, p < 0.001), operation time (AOR 1.45, CI: 1.19-1.76, p < 0.001), female gender (AOR 2.16, CI: 1.06-4.40, p = 0.034) and lower self-rated health (AOR 0.63, CI: 0.41-0.99, p = 0.044).

**Conclusions:**

Lower happiness and self-rated health may influence the outcome of cardiac surgery. Therefore, these variables should be assessed in patients.

## Background

Coronary artery bypass grafting (CABG), with or without heart valve surgery is a safe and effective intervention for patients with symptomatic coronary artery disease. Although surgical techniques and perioperative care continue to improve, there is still a significant risk of death or major morbidity after surgery. Therefore, the accurate prediction of risk plays a pivotal role in clinical practice. The widely used EUROSCORE risk assessment system covers only medical variables; however, medical factors alone do not fully explain short- and long-term physical health outcomes, including postoperative length of hospital stay, recurrent cardiac events and CABG-related mortality [1]. Prolonged intensive care unit (ICU) stay does not only mean a significant use of hospital resources [2] but is also a risk factor for death within 60 days of hospital discharge [3]. Despite the increasing recognition of psychosocial factors in cardiovascular morbidity and mortality, CABG patients do not routinely receive psychosocial screening. However, identification of preoperatively existing psychosocial risk factors might improve clinical risk stratification, decision-making and the ultimate success of surgery. Although depressed and anxious mood states [4, 5] are perhaps the most strongly established psychosocial variables linked to cardiovascular outcomes, other characteristics may also be important. For example, we found that poor social support and self-rated health predict poor health outcomes in CABG patients [6].

The purpose of this study was to investigate the possible association of prolonged ICU and hospital stay following CABG and valve surgery with psychosocial factors after adjustment for medical factors. Little evidence is available about potential predictors. Moreover, psychosocial variables that are possible predictors of prolonged ICU and hospital stay have not yet been adequately controlled for biomedical and other psychosocial factors. Furthermore, identification of these predictors may help to assess risks for extended ICU and hospital after cardiac surgery.

## Methods

### Participants

Six hundred forty-four consecutive patients awaiting elective CABG, combined CABG/valve, or valve surgery only at Gottsegen Hungarian Institute of Cardiology were eligible to participate between November 1, 2006 and October 31, 2007. We considered all patients eligible to participate regardless of age, sex, cardiac condition or on- versus off-pump surgical procedures. The only exclusion criterion was severe psychological/psychiatric comorbidity, because these patients were unable to answer our questionnaires. The patients in the study were admitted to the surgical ward at least 2 days before surgery.

After admission, we invited patients to participate in our study. Patients completed baseline questionnaires 1.56 days (standard deviation (SD) = 0.7) before surgery. Tests were fulfilled by the patients*.* Two hundred sixty-seven (41.5%) patients agreed to participate and provided informed consent. Surgery was cancelled for 4 patients and 3 patients had non-CABG surgery (2 pericardectomy, 1 porcelain aorta). Nineteen patients were unable to complete the psychological tests for various reasons (e.g., mental disabilities and dysgraphia). If patients refused or were unable to participate (n = 370; 57.5%), we used only their medical data from the institutional database. The participation of patients with neuropsychiatric disorders was very low, only 2 patients had treated depression, 1 patient had major depressive psychosis, 2 patients had preoperative stroke. In the postoperative period, 10 patients (3.7% of 267) had neurological complications, and at 9 patients were started antidepressant treatment in the postoperative period*.* The Institutional Medical Ethics Committee approved this study, file number: EKB 625-1/2006.

Written informed consent was obtained from every patient for the publication of this report.

### Clinical factors

We assessed a wide range of demographic, medical and psychosocial factors as potential predictors of length of ICU and hospital stay. Medical factors included previous myocardial infarction, previous CABG or valve surgery, history of arrhythmia, congestive heart failure, diabetes mellitus, hypercholesterolemia, cerebrovascular disease, chronic renal insufficiency, hypertension, chronic obstructive pulmonary disease, gastrointestinal disease, peripheral arterial disease, pulmonary hypertension, stroke and previous psychiatric hospitalization. Detailed definitions of these variables can be accessed at the Society of Thoracic Surgeons’ homepage [7]. Also smoking was evaluated, we categorized patients into two groups: those who quitted smoking before operation (previously smoking group) and those who did not (right before operation group). In addition, we examined specific aspects of the surgical procedure. Finally, we noted the responsible surgeon, whether an intraoperative intraaortic balloon pump was used and whether a heart-lung machine was used (i.e., an on/off pump procedure). Continuous variables from patient medical charts included the number of grafts, cardiopulmonary bypass time, aortic cross clamp time and total duration of surgery (in minutes).

The Society of Thoracic Surgeons Database [8] defines the key outcomes of postoperative complications, such as permanent stroke (new-onset cerebrovascular accident persisting for more than 72 hours), reoperation for any reason, serious infection (e.g., positive blood culture, deep sternal wound infection and catheter-related infection), prolonged mechanical ventilation (ventilation support for more than 48 hours), renal failure requiring dialysis and myocardial infarction. A diagnosis of acute heart failure required the use of an intra-aortic balloon pump or continuous intravenous inotropic support for at least 48 hours. These postoperative complications were added together and the number of postoperative complications was created as a continuous variable (postoperative complication score). We also measured the duration of ICU and total hospital stay.

### Potential covariates

We investigated eight demographic variables (age, sex, marital status, current living/working arrangements, number of children and siblings, and years of education completed) as potential covariates. We used several validated psychosocial tests with Cronbach’s alfa for study population. Self-rated health (SRH) was measured with the question: “How do you rate your health in general?” There were five possible responses: very good, good, fair, poor and very poor (1 = very poor, 5 = excellent) [9]. Cronbach’s alpha for the studied population is not available as it was single question.

The question “How happy are you in general?” assessed happiness. The answer was rated from unhappy (0) to happy (10). The same question was used in Hungarostudy [10].

General satisfaction was measured by a single question “How satisfied are you?”

The trait scores of the Spielberger State-Trait Anxiety Inventory (STAI-T) characterized the patients’ anxiety symptoms. STAI-T scores reflect relatively enduring traits with regard to anxiety. STAI-T scores range from 0–70; scores of 0–48 indicate no psychiatric disorder; 48–52 indicate mild/transient symptoms and scores above 52 suggest the presence of a psychiatric disorder. The validity and reliability of the full version of STAI-T has been well documented in the Hungarian population [11, 12]. Cronbach’s alpha was calculated for the studied population as 0.81. The STAI-T has been used in more than 3000 studies and has been translated into over 30 languages. Since Spielberger’s review more than 1000 articles contain the test according to recent PubMed search, suggesting the measure continues to be very popular in psychological research. However, the test has been criticized for its inability to adequately discriminate between anxiety and depression symptoms [13].

The full version of the Beck Depression Inventory (BDI) is a self-report checklist that assesses cognitive, affective, behavioural and physiological symptoms of depression. Scores from 0–9 are normal, 10–18 indicate mild to moderate depression, 19–25 suggest moderate to severe depression and scores above 26 indicate severe depression [14]. Cronbach’s alpha was calculated for the studied population as 0.80. In case of BDI, the way the instrument is administered can have an effect on the final score. If a patient is asked to fill out the form in front of other people in a clinical environment, for instance, social expectations have been shown to elicit a different response compared to administration via a postal survey [15]. In participants with concomitant physical illness the BDI’s reliance on physical symptoms such as fatigue may artificially inflate scores due to symptoms of the illness, rather than of depression [16].

Cronbach’s alfa provides a measure of the internal consistency of a test or scale; it is expressed as a number between 0 and 1 [17].

### Outcome assessment

The clinical outcomes were length of ICU and hospital stay (i.e., the number of days from the date of surgery to the date of ICU discharge). We defined extended postoperative ICU stay as a length of at least 3 days for patients who were alive at discharge. The mean ICU stay was 5.7 days (median = 1 day 95% C.I.: 1–6 days) with a skew of 4.6. Previous literature has transformed or dichotomized skewed length of stay data [18]. There is no consensus in the definition of a prolonged ICU stay; it extended from 1 to 10 days depending on the authors’ choice. In accordance with Bucerius et al. who defined extended ICU stay as ≥3 days in the conviction that it includes almost all patients suffering postoperative complications [19] we chose to dichotomize this variable at 3 days because this definition identified a clinically meaningful subset of patients (14.3%) with a higher incidence of post-procedure events.

### Statistical analyses

We described the data in terms of the mean and SD or the median and the interquartile range for continuous variables, and as frequencies and percentages for categorical variables. We used different variables from EUROSCORE and those showing p < 0.20 in the univariate analysis were entered into a multivariable logistic regression model (e.g. gender, COPD, diabetes, pulmonary hypertension).

To create a base model, a multivariate analysis used medical record predictors including age, sex, preoperative clinical variables and intraoperative surgical factors. To minimize collinearity, we used the length of the operation instead of cardiopulmonary bypass time and aorta cross-clamp time, because they were highly correlated.

Next, we developed a patient survey model using patient survey data predictors (socioeconomic questions and psychosocial measures) to determine the predictors that were associated with extended ICU stay and hospital stay after adjusting for the base variables. In the patient survey model, univariate analysis analyzed the relationship between extended ICU stay and the psychosocial test scores.

The multivariable model combined the base model with the patient survey model to determine the psychosocial predictors that were independently related to extended ICU stay after controlling for sociodemographic and medical predictors. Again, we entered all predictors (p < 0.2) into the model. We assessed the fit of the logistic models using the Hosmer-Lemeshow Goodness-of-Fit test. Bootstrapping method was used to examine the discriminating power of the logistic model using the area under the receiver operating characteristic (AUC-ROC) curve. We performed all analyses using SPSS 16.0 (SPSS Inc., Chicago, IL).

## Results

Of the 644 eligible patients, we analyzed the data of 267 (41.5%). The mean age was 60.3 years (SD: 8.9 years) and 73% of the patients were male. The most common preoperative conditions were hypertension, hyperlipidemia, previous myocardial infarction, diabetes and arrhythmia (Table 1).

**Table 1:** **Pre- and perioperative characteristic of participants**

	N = 267	
	Number/median/mean	%/(IQR)/SD
**Preoperative factors**		
Age (years)	60.3	8.9
Male	194	73%
Female	73	27%
Body weight (kg)	81.9	14.5
BMI (kg/m2)	27.9	4.3
**Medical history**		
Hypertension	204	76.4%
Diabetes	66	24.7%
Myocardial infarction	84	31.5%
Arrhythmia	62	23.2%
Hyperlipidemia	156	58.4%
Kidney failure	25	9.3%
COPD	37	13.8%
Gastrointestinal disease	57	21.3%
NYHA III/IV	30	11.2%
Atrial fibrillation	39	14.6%
Stroke	8	3.0%
Peripheral arterial disease	23	8.6%
Pulmonary hypertension	17	6.4%
**Perioperative factors**		
Valve	101	37.8%
CABG	174	61.4%
Complex procedure	21	7.9%
Operation time	133	(110–160)
Cardiopulmonary bypass time	37	(0–71)
Off-pump procedure	147	55.1%
Surgeon A	24	9.8
B	13	5.9
C	39	15.1
D	13	4.8
E	87	26.1
F	37	17.3
G	13	6.8
H	33	11.1
I, J, K	9	2.6
**Postoperative complication**		
Sepsis	7	2.6%
Death	4	1.5%
Dialysis	6	2.2%
Intra-aortic balloon pump	11	4.1%
Mechanical ventilation more than 48 hours	12	4.5%
Bleeding	20	7.5%
Acute myocardial infarction	7	2.6%
Sepsis	9	3.4%
Liver failure	9	3.4%
Multiple organ failure	5	1.9%
Neurological disorder	10	3.7%
Hospital days	9.5	6.2

IQR: Interquartile range; SD: standard deviation; BMI: body mass index; COPD: Chronic obstructive pulmonary disease; NYHA: New York Heart Association; CABG: Coronary artery bypass grafting; ASA: American Society of Anesthesiologists risk score. Data are described as the mean and standard deviation (SD) or the median and interquartile range (25th–75th percentile) for continuous variables and as counts and percentages for categorical variables.

The median length of ICU stay was 1 day (range = 0-46 days). Those who spent 3 or more days in the ICU were treated for 839 days of the total of 1,437 (58.4%).

The mean hospital stay was 9.55 days (SD: 6.22) and 62 patients (23.3%) spent more than 10 days in the hospital. There was no significant difference in terms of age and BMI between the two groups; patients with long hospital stay had longer operation time, longer ICU stay and more complications in the ICU. These patients were more likely to have preoperative arrhythmia, COPD, heart failure functional class III/IV, and they had more postoperative complications, like need for IABP, respiratory failure, neurological disorder (any new neurological deficit after surgery), sepsis and need for antidepressant use. There were more female patients among them (Table 2).

**Table 2:** **Hospital stay**

	Hospital stay <10 days n/mean (%/SD)	Hospital stay ≥10 days n/mean (%/SD)	p-value
Age	59.8 (9.3)	62.1 (7.2)	0.39
Female	49 (23.9%)	24 (38.7%)	**0.02**
BMI	28.0 (4.2)	27.5 (4.5)	0.43
Hypertension	160 (78.4%)	44 (71.0%)	0.22
Diabetes mellitus	52 (25.4%)	14 (22.6%)	0.66
Arrhythmia	41 (20.0%)	21 (33.9%)	**0.02**
Hyperlipidemia	122 (59.5%)	34 (54.8%)	0.51
Renal failure	18 (8.8%)	7 (11.3%)	0.55
COPD	19 (9.3%)	18 (29.0%)	**<0.001**
NYHA III/IV	14 (6.8%)	16 (25.8%)	**<0.001**
Stroke	6 (2.9%)	2 (3.2%)	0.90
**Perioperative factors**			
Operation time	134.0 (40.2)	166.9 (74.6)	**0.001**
Off pump	118 (80.0%)	29 (19.7%)	0.14
CABG	137 (78.7%)	37 (21.3%)	0.30
Valve surgery	72 (71.3%)	29 (28.7%)	0.10
**Postoperative factors**			
ICU days	1.46 (3.00)	5.54 (9.16)	**0.001**
Respiratory failure	4 (2.0%)	8 (12.9%)	**<0.001**
Bleeding	12 (5.9%)	8 (12.9%)	0.07
Neurological disorder	3 (1.5%)	7 (11.3%)	**<0.001**
Sepsis	2 (1.0%)	5 (8.1%)	**0.002**
Antidepressant therapy	3 (1.5%)	6 (9.7%)	**0.002**

SD: standard deviation; BMI: body mass index; COPD: Chronic obstructive pulmonary disease; NYHA: New York Heart Association; CABG: Coronary artery bypass grafting; IABP: intraaortic balloon pump.

Bold values represent statistical significance.

We also investigated the relationship between the psychosocial tests. BDI and STAI-T scores correlated to each other (r: 0.24, p **<** 0.001) BDI and happiness exhibited inverse correlation (r:-0.21, p < 0.001). Satisfaction and happiness correlated strongly (r: 0.72, p < 0.001) and satisfaction and STAI-T correlated inversely (r: -0.47, p < 0.001). Similarly, there was an inverse correlation between happiness and STAI-T scores (r: -0.51, p < 0.001). SRH showed no correlation with any tests.

Patients with ICU stay equal or longer than 3 days had lower self-rated health and lower happiness scores, but higher BDI scores. Smoking before surgery, level of education and living alone did not influence the length of ICU stay. After controlling for medical and sociodemographic factors, lower self rated health, lower happiness, longer operation time, higher NYHA stages and the occurrence of severe COPD were independently associated with long ICU stay (Table 3). We adjusted for medical factors including age, gender, NYHA class, diabetes, complex surgery, operation time. The c-index of the multivariate model was 0.72 and the Hosmer-Lemeshow chi-square test = 5.3, p = 0.72. Adding psychosocial factors (BDI, STAI-T, somatic severity, self rated health and happiness) to the model improved the c-index to 0.77.

**Table 3:** **Multivariable model for ICU stay**

	AOR	95% CI		p-value
COPD	3.34	1.30	8.56	**0.012**
NYHA	2.99	1.06	8.42	**0.038**
Operation time (30 min)	1.41	1.14	1.75	**0.001**
Self-rated health (points)	0.60	0.36	0.99	**0.045**
Happiness (points)	0.84	0.71	0.98	**0.032**

OR: odds ratio; CI: confidence interval; AOD: adjusted odds ratio; COPD –chronic aspecific pulmonary disease; NYHA: New York Heart Association heart failure severity class. P < 0.05 indicates significance and is highlighted bold.

Long hospital stay (longer than or equal to 10 days) was associated with NYHA severity, COPD, longer operation time and female gender. Among psychosocial factors, self-rated health remained a predictor (Table 4). Adding psychosocial factors (BDI, STAI-T, somatic severity, self rated health and happiness) to the final multivariate model improved the c-index from 0.74 to 0.76 and the Hosmer-Lemeshow chi-square test = 6.8, p = 0.55.

**Table 4:** **Multivariable model for hospital stay longer than 10 days**

	AOR	95% CI		p-value
COPD	4.56	1.95	10.67	**<0.001**
NYHA	6.76	2.57	17.79	**<0.001**
Operation time (30 min)	1.45	1.19	1.76	**<0.001**
Female gender	2.16	1.06	4.40	**0.03**
Self-rated health	0.63	0.41	0.99	**0.04**

OR: odds ratio; CI: confidence interval; AOD: adjusted odds ratio; COPD chronic obstructive pulmonary disease; NYHA New York Heart Association heart failure severity class. P < 0.05 indicates significance and is highlighted bold.

## Discussion

We have found that long operation time, high NYHA levels and severe COPD were significantly associated with ICU stay equal to or longer than three days after cardiac surgery. After adjusting for medical factors, lower self-rated health and low level of happiness were also independently associated with length of ICU stay. Long hospital stay was associated with the same clinical factors and female gender but only self-rated health predicted it among the psychosocial factors after adjusting for depression and anxiety.

Since the in-hospital mortality after cardiac surgery is generally low (between 2% and 6.6% among studies) [20, 21], several studies have been attempted to investigate the role of medical and psychosocial variables in the length of ICU or hospital stay or postoperative complications. Many authors have tried to develop a scoring system to better predict extended ICU stay and obtained different results. These medical factors included several risk factors for cardiac surgery (congestive heart failure, sepsis, renal failure, respiratory insufficiency, and cardiac arrest), major cardiovascular events or both [22, 23]. Risk scoring algorithms exclusively for the prediction of ICU stay can emphasize objective, patient-specific estimation of early risk and short-term prognosis. However, many of these algorithms have not been validated in multi-institutional, international studies and in different patient populations. Interpretation of these algorithms can be difficult, since all risk systems evaluate different patient populations and types of cardiac surgical interventions [24].

In our study, severe COPD, cardiac failure and long operation time were associated with prolonged ICU stay. Patients with preoperative CHF often have pulmonary edema, which deteriorates the ventilation/blood flow ratio, thus resulting in postoperative hypoxemia [25]. In addition, long-term respiratory muscle overwork may result in postoperative respiratory failure [26]. In a study about need for re-intubation and therefore extended ICU stay, preoperative congestive heart failure and severe COPD were important associated factors following CABG. The investigators observed small airway obstruction and larger physiological dead space in patients suffering from COPD. Furthermore, CABG surgery with cardiopulmonary bypass reduced the volumes and capacities of the lung [27]. The association between lower left ventricular ejection fraction and longer ICU stay and hospitalization was also observed by other authors [22, 28]. Consistent with previous findings, female gender was found to be an independent risk factor. Although the explanation is controversial [29, 30], it has been suggested that a greater mortality among women may be a reflection of their greater severity of illness, increased morbidity at presentation, smaller body surface area, and smaller coronary vessels [31].

This study’s main finding is that low self-rated health and lower happiness significantly predicted prolonged ICU stay. Low self-rated health was also associated with longer hospitalization. Assessment of both variables before surgery is a quick and non-invasive, easy tool for clinicians, since each consists of one question and takes only short time to answer. Self-rated health was associated with mortality, even after controlling for a wide range of health measurements and mortality risk factors in accordance with previous findings [32–34]. In our study, self-rated health was an independent but not an exclusive predictor of longer ICU and hospital stay. In a previous study, we found that lower self-rated health was associated with an increased risk of major cardiac and cerebral events five years after cardiac surgery, even after adjusting for biomedical and psychosocial factors [6].

Predicting whether a critically ill patient will need a long ICU and hospital stay (thus having a greater risk of morbidity and mortality) remains difficult and the advantages of identifying patients who do not benefit from ICU treatment are evident. SRH is an individual and subjective conception that is related to death, and builds a connection from the social and psychological approach to the biological world. Therefore the answer to the SRH question may summarize the dimensions of health that are most important and determinant for each individual [35]. SRH has been described as one of the most important health outcomes available and recommended as a tool for disease risk screening, as an outcome indicator in the primary care, and standard part of clinical trials [36]. Therefore, we recommend that self-rated health score should be a complementary outcome measure in clinical trials and clinical practice. Besides its important predictive value, SRH provides possibilities for intervention. In an interventional study about the effect of telephone based counselling on self rated health of cardiac patients the authors found that the intervention made a significant improvement on patient SRH among distressed patients hospitalized for cardiac disease. The authors suggested that this treatment might be an effective additional disease management program [37].

Negative emotional states (e.g., depressed and anxious mood and anger) are proven risk factors for cardiovascular disease [38]; however, much less is known about the association between positive emotional states (e.g., happiness and optimism) and cardiovascular health. Steptoe et al. have suggested that positive emotions may have a direct and beneficial effect on physiological processes including those involving neuroendocrine, inflammatory, immunological and cardiovascular systems [39]. The association between positive psychological well-being and mortality could be mediated in part via behavioural pathways. For example, positive dispositions are related to predictors of prolonged survival, such as not smoking, exercising regularly, reduced alcohol consumption, and better sleep quality. Psychologically balanced persons might have increased adherence to medical regimens as inverse associations between adherence and depression have been described. However, the protective effect of positive emotions on mortality in healthy population studies persisted even after fully controlling for behavioural covariates, suggesting that other pathways may also be involved. Direct physiological pathways might also contribute to associations. Positive psychological well-being could alter the disease susceptibility of people via the attenuation of sympathetic nervous system activity and the enhancement of parasympathetic activation. Positive affects may reduce stress-induced elevations of inflammatory and coagulation factors, such as fibrinogen and interleukin-6, which are crucial in cardiovascular diseases, and reduce vulnerability to infectious illness. Positive psychological well-being was associated with reduced cardiovascular mortality in healthy population studies, with a near significant effect in patients with established cardiovascular disease [40]. Optimism was associated with better recovery from CABG surgery within 6 months [41]. We showed that a lower rating of happiness significantly predicted longer ICU stay. Low level of positive emotions does not necessary mean depression and vice versa, since we found only moderate correlation between depression and happiness; however, the inverse correlation was stronger between anxiety and happiness.

Anxiety, which is an established risk factor for cardiac mortality [42, 43] increases sympathetic activity with its known cardiovascular adverse affects (e.g. abnormally increased production of catecholamines, which can result in increased myocardial oxygen demand due to elevations in heart rate, blood pressure, and the rate of ventricular contraction) [44], so it has the opposite effect as happiness. The relationship between a higher level of depression (BDI > 10 points) and longer post-operative length of hospital stay in univariate analysis is consistent with the relationships shown in previous cardiac research [45–49], however this could not be detected in multivariable analysis using continuous variables and adjusted for postoperative complications. It can be speculated that the role of depression need a longer period to cause adverse event after cardiac surgery like 5 year or after [50].

This study has certain limitations; the most important one being that 42% of the population refused participation and small sample size. Non-participants were more likely to be female and had higher incidences of hypertension, diabetes, arrhythmia, gastrointestinal diseases and stroke and higher risk scores. Thus, the present results may actually underestimate the importance of SRH and happiness since these may be thought to have been lower in non-participants. Psychological factors predicting length of ICU stay may have been somewhat biased by institutional factors (number of beds, surgery case load, insurance, etc.). Therefore, our sample may not have been representative of the entire cardiac surgery population. Our single-center findings need further investigation in a larger, multicentre population. Furthermore, patients with severe psychological/psychiatric comorbidities could not be enrolled in our study, due to inability to fill out questionnaires. We enrolled patients who underwent CABG surgery, valve surgery or both. Not differentiating between these types of surgeries in our analyses may have influenced our results, although previous reports did not find significant differences with regard to mortality and morbidity [51]. Interpretation and integration of our results can be difficult, since all prior ICU and hospital and prediction studies evaluate different patient populations and types of cardiac surgical interventions. We used a rather extensive set of biomedical and psychosocial variables to cover as much predictors as possible in a mixed cardiac population. We could not rule out that the preoperative scores of individual tests were influenced by acute changes in the health status or conversely long duration of a disease modified the score. Finally, we could not establish causal relationships because our study was purely observational, e.g. we had only one measurement point.

All questionnaires used share the same problems as other self-report inventories, in that scores can be easily exaggerated or minimized by the person completing them. However, they are widely used in clinical studies, generally among similar circumstances (e.g. before and after difficult life-situations, like in this case before and after surgery) and accepted in psychological research.

Self-rating about general health is influenced by several clinical factors, e.g. it is a better predictor of mortality among men than among women, which suggests men evaluate their health status differently. Obese patients may incorporate their information about health risk associated with obesity in their assessments independent from signs and symptoms. Individuals with high cholesterol are also influenced by the diagnosis, although the disorder is asymptomatic [52].

A study about pre- and postoperative depression revealed that depressed individuals tend to be physically and emotionally vulnerable to the stresses of surgical interventions. The catastrophic thinking before operation could heighten depressive symptoms due to the uncertain outcomes (i.e. survival vs. death). Conversely, improvement in physical functioning seen in many patients following CABG may have the added effect of elevating mood [48].

## Conclusion

We found that assessing particular psychosocial factors might predict the likelihood of longer ICU and hospital stay. Long hospital stay was associated only with self-rated health after adjusting for medical variables. Lower scores of self-rated health and happiness were independently associated with longer ICU stay after adjusting for the most relevant medical factors. These tests are easy to perform, and do not take long time. Measures of self-rated health, happiness, and depressed and anxious moods have not previously been studied simultaneously; thus, this study may fill a gap in the field of emotional factors and chronic stressors of cardiac surgery.

## Abbreviations

AOR: Adjusted odds ratio
ASA: American Society of Anesthesiologists risk score
BDI: Beck depression inventory
BMI: Body mass index
CABG: Coronary artery bypass grafting
CI: Confidence interval
COPD: Chronic obstructive pulmonary disease
CU: Intensive care unit
IQR: Interquartile range
N: Number
NYHA: New York Heart Association
OR: Odds ratio
ROC: Receiver operating characteristic
SD: Standard deviation
SRH: Self-rated health
STAI-T: Spielberger state-trait anxiety inventory.
